# Giant right atrium in a child with dilated cardiomyopathy: A case report

**DOI:** 10.3389/fcvm.2023.1083188

**Published:** 2023-03-15

**Authors:** Benzhen Wang, Guangsong Shan, Zhen Bing, Qi Zhang, Quansheng Xing, Zipu Li

**Affiliations:** ^1^ Heart Center, Qingdao Women and Children’s Hospital, Qingdao University, Qingdao, China; ^2^ Department of Radiology, Qingdao Women and Children’s Hospital, Qingdao University, Qingdao, China

**Keywords:** heart failure, right atrium, cardiomyopathy, pediatrics, cardiac surgery

## Abstract

Dilated cardiomyopathy (DCM) is one of the leading causes of heart failure in children with diverse clinical characteristics. To date, DCM with a giant atrium as the first manifestation is rare and has not been reported in previous literature. We report a case of a male infant born with a significantly enlarged right atrium. Due to worsened clinical symptoms and the risk of arrhythmias and thrombosis, we performed the surgical reduction of the right atrium. Unfortunately, DCM and a progressive re-enlargement of the right atrium appeared during midterm follow-up. The mother's echocardiogram also suggested DCM, and the patient was eventually considered for a diagnosis of familial DCM. This case may expand the clinical spectrum of DCM and reminds us of the importance of good follow-up of children with idiopathic dilatation of the right atrium.

## Introduction

Dilated cardiomyopathy (DCM) is one of the leading causes of heart failure in children with complex etiologies and diverse clinical manifestations (1). Marked enlargement of the right atrium (RA) as the initial presentation of DCM has not been reported. Herein, we report an infant born with a giant right atrium and developed DCM during midterm follow-up.

## Case report

We report the case of a male infant born from cesarean section after a normal pregnancy, with an Apgar score of 10/10 at 1st and 5th minutes, respectively. His mother was a 28-year-old, healthy, Gesta 2 para 2 woman who was presented for an obstetric ultrasound at 30 weeks of gestational age. An enlargement of the right atrium was noted. No other fetal anatomic abnormalities were detected. After birth, the case was admitted to the cardiac intensive care unit at the Qingdao Women and Children's Hospital. Family medical history was normal.

**Figure 1:**
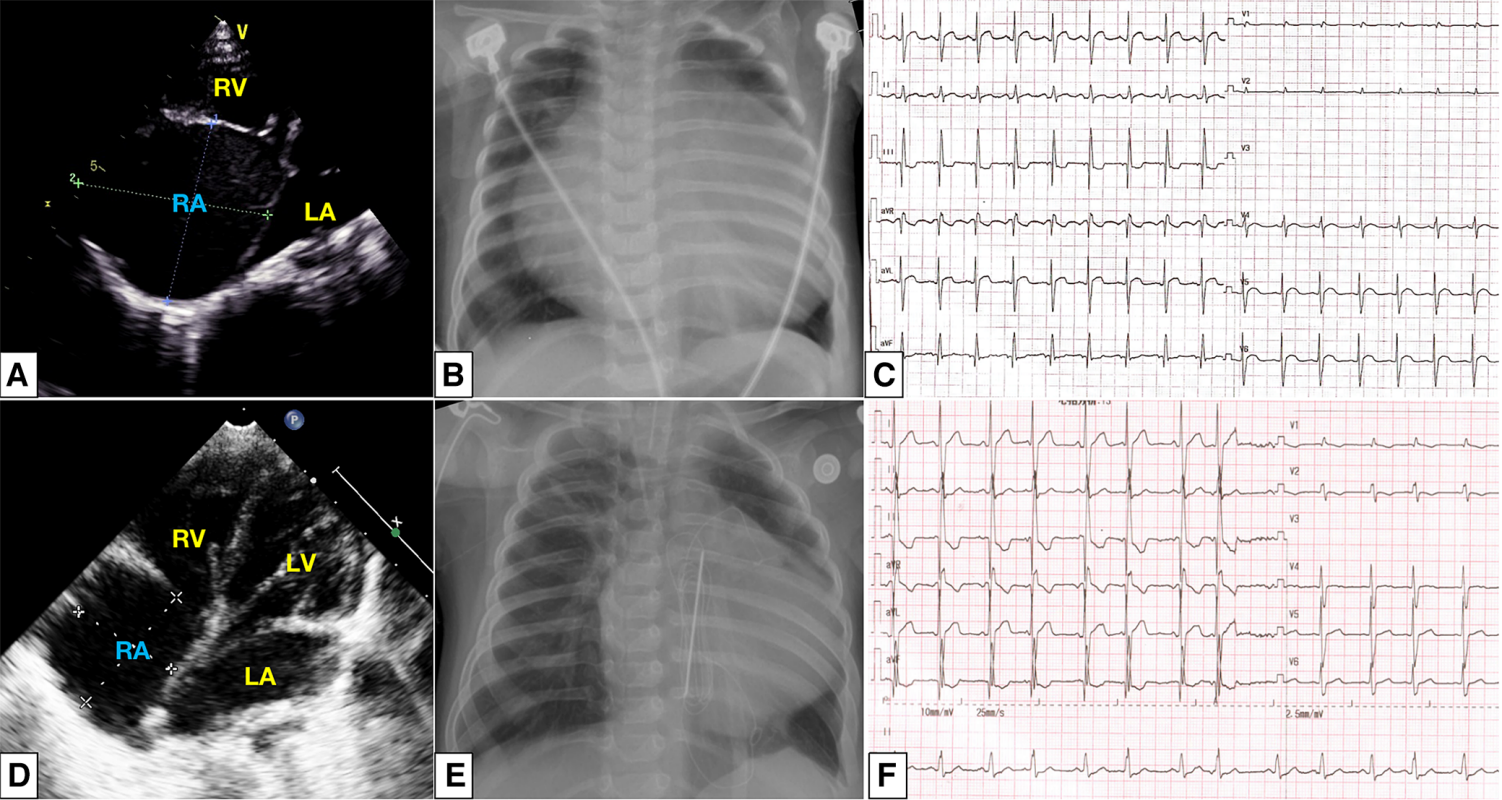
At initial presentation: a dilated right atrium was noted in apical four-chamber view (**A**); chest radiography showed cardiomegaly with a CTR of 80% (**B**); electrocardiograph showed sinus tachycardia, right axis deviation, and incomplete right bundle branch block (**C**). After surgical reduction, a slightly enlarged right atrium and intact atrial septum (**D**), CTR decreased to 0.61 (**E**), and the atrial premature beat was detected (**F**). CTR, cardiothoracic ratio;

Heart rate, respiratory rate, blood pressure, and oxygen saturation on room air were normal on admission. No cyanosis, heart murmurs, wheezing, or hepatomegaly were present. Blood gas analysis, routine test, and liver and renal function were normal. The patient's and his mother's erythrocyte sedimentation rate, antinuclear antibodies, and extractable nuclear antigens were negative. N-terminal pro-brain natriuretic peptide reached 2,035 pg/mL (reference ranges <125 pg/mL). Transthoracic echocardiogram (TTE) showed a giant RA (50 mm × 48 mm) with normal right and left ventricular volumes, a 15 mm secundum atrial septal defect (ASD), the tricuspid annulus was 14 mm, slight tricuspid regurgitation, a pulmonary arterial systolic pressure (PASP) of 45 mmHg, and left ventricular ejection fraction (LVEF) of 65% (Figure 1A). The chest x-ray showed an enlarged heart, and the cardiothoracic ratio (CTR) reached about 0.80 (Figure 1B). Electrocardiograph showed sinus tachycardia, right axis deviation, and right bundle branch block (Figure 1C). Computed tomography angiography indicated abnormalities similar to TTE. After extensive discussion, the initial diagnosis we considered was idiopathic dilatation of the right atrium (IDRA). The patient was discharged and followed up regularly every month in the clinic.

**Figure 2:**
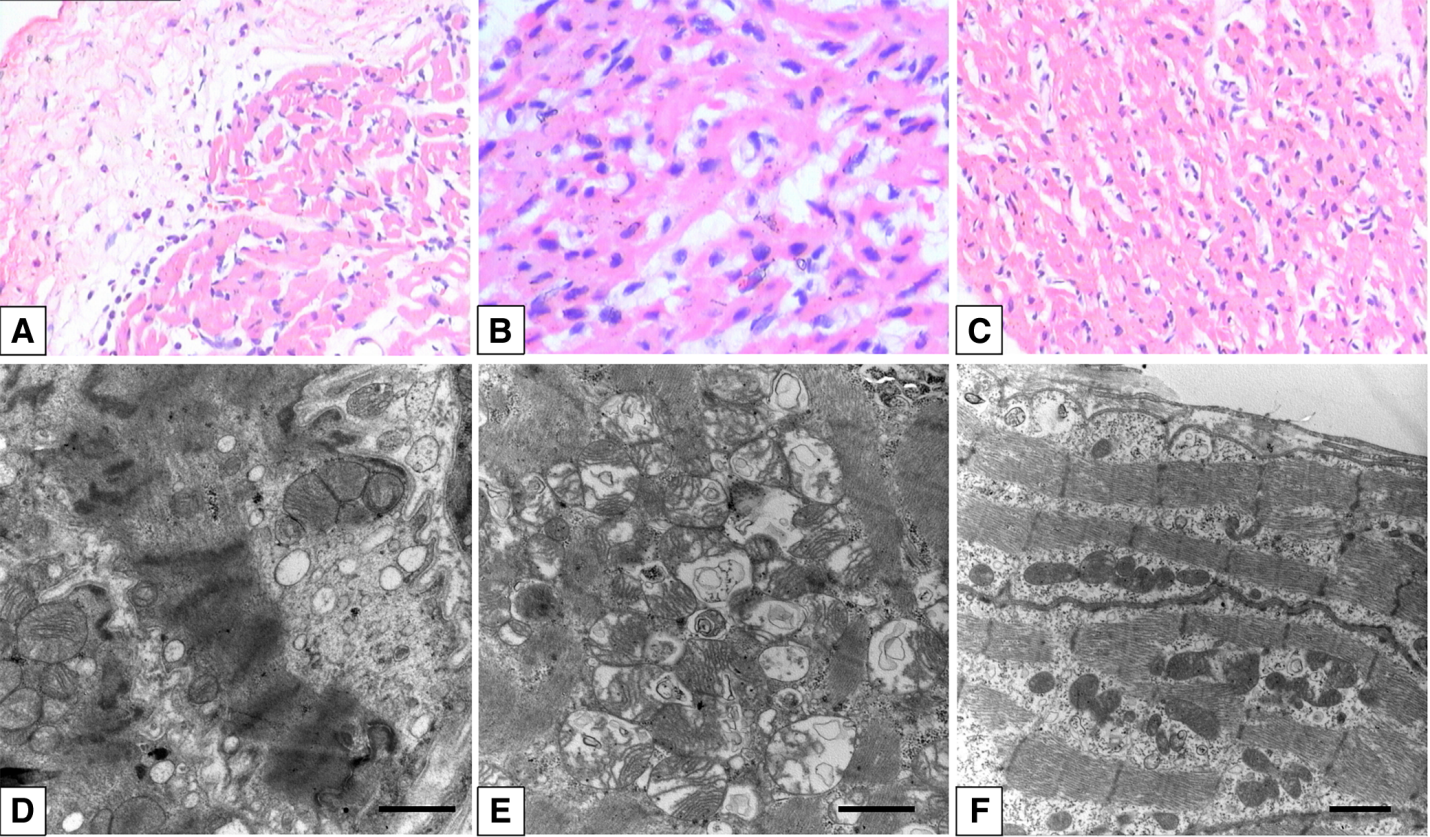
H&E staining showing the pathological changes in myocardial tissues of the right atrium (**A**), right ventricle (**B**), and left atrium (**C**). TEM shows damage to myofibrils and mitochondria in the right atrium (**D**), right ventricle (**E**), and left atrium (**F**). Scar bar: 1 µm. Magnification: ×25,000 and ×20,000. TEM, transmission electron microscopy.

**Figure 3:**
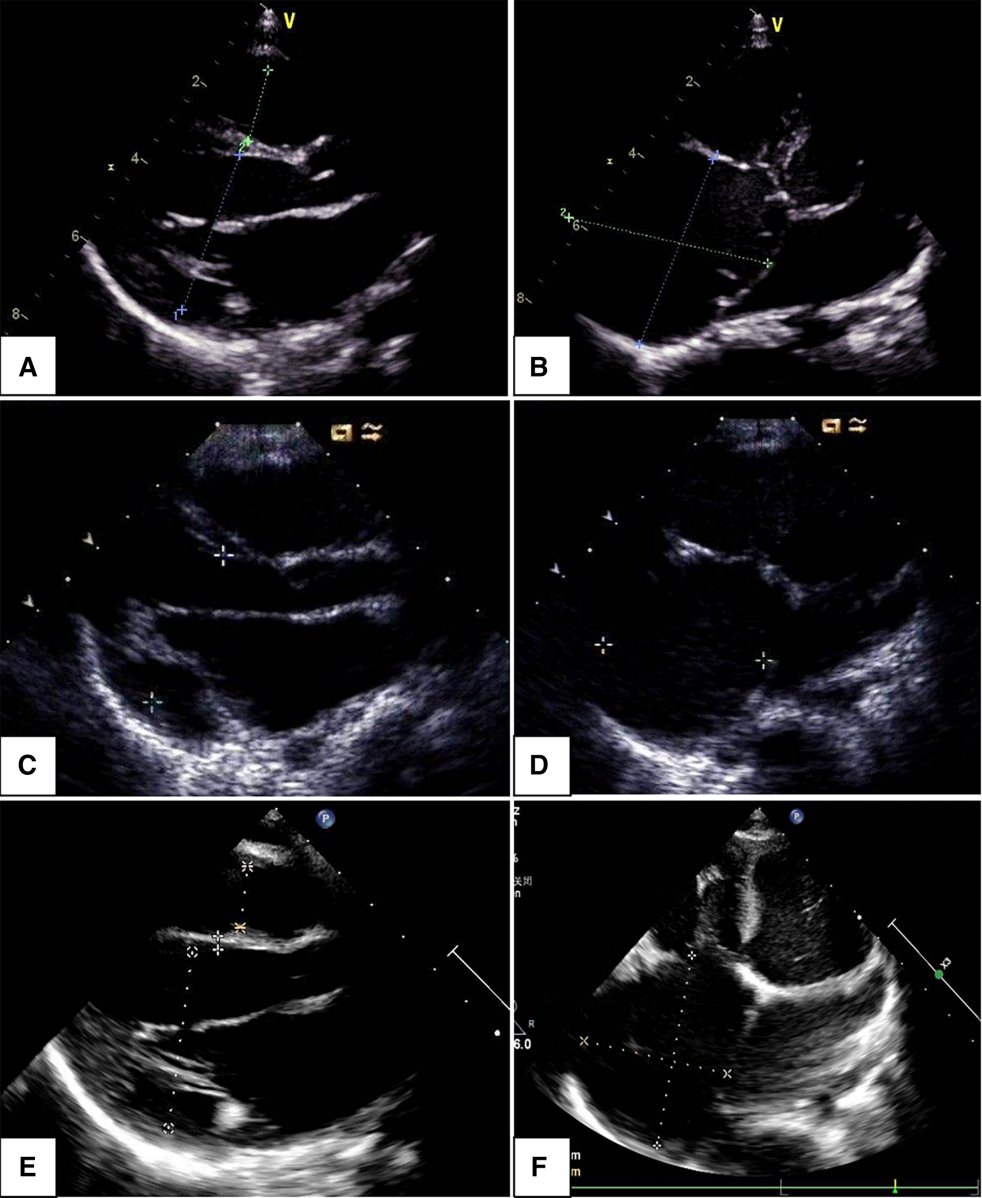
Changes in echocardiogram with follow-up time: parasternal long axis view indicated left ventricular end-diastolic dimension increased within 1, 2, and 5 years (**A**,**C**,**E**); apical four-chamber view showed an enlarged right atrium, the changes were not significant within 1, 2, and 5 years (**B**,**D**,**F**).

**Figure 4:**
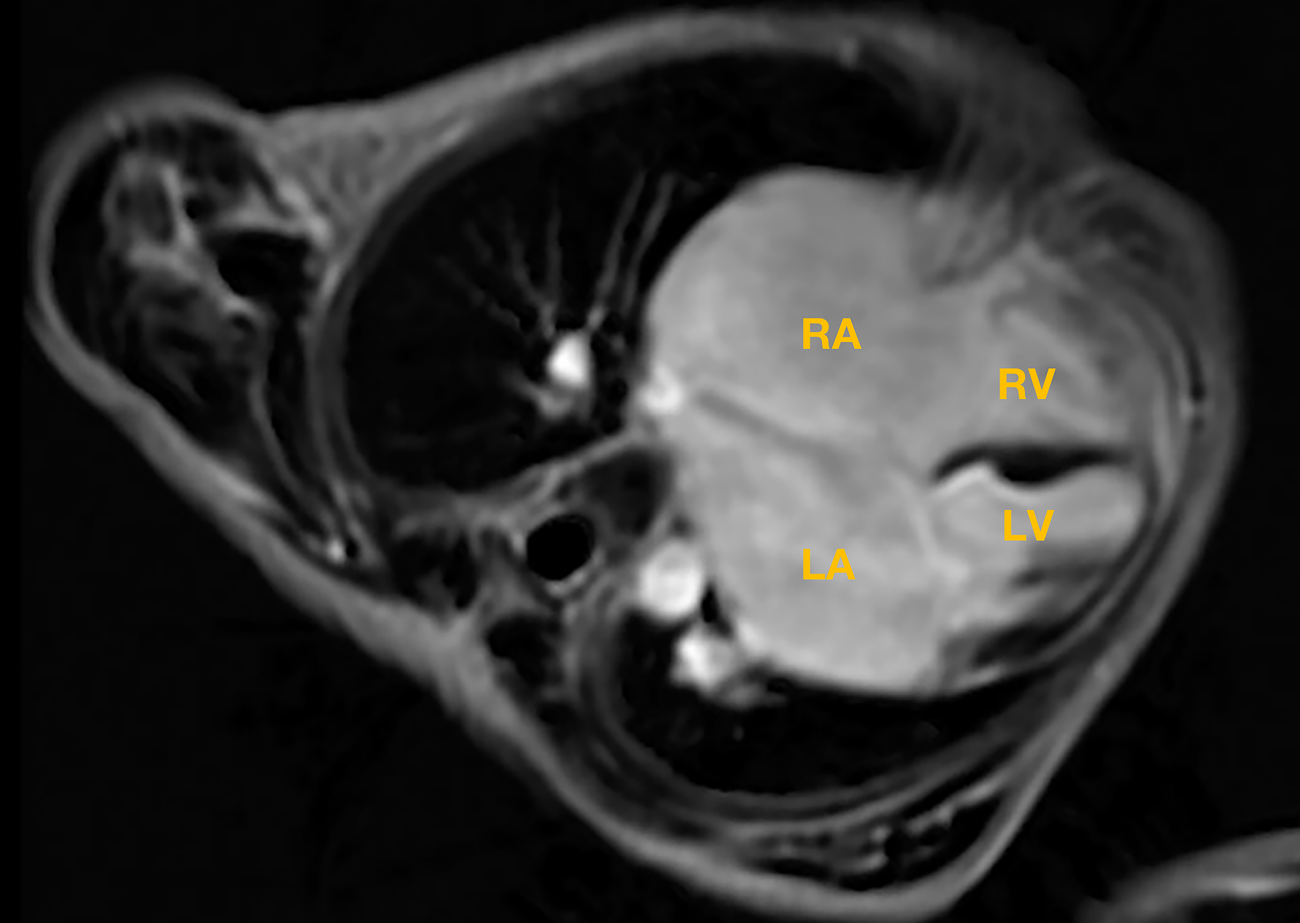
Cardiac magnetic resonance showed an enlarged right atrium, left atrium, and left ventricle; no typical manifestations of ventricular myocardial fibrosis were detected. RA, right atrium; LA, left atrium; RV, right ventricle; LV, left ventricle.

Clinical symptoms worsened during the patient's follow-up, such as feeding difficulty, shortness of breath, and growth retardation. Considering the possibility of compression caused by the enlarged RA and the risk of arrhythmias and thrombosis, we performed the surgical reduction of RA at 3 months with consent obtained from the parents. Transesophageal echocardiography and intraoperative findings confirmed the diagnosis of giant RA and ASD. Resection of extensive RA wall and atrioseptopexy were performed. At surgery, biopsies of the RA wall, right ventricle (RV) and left atrium (LA) revealed unspecific inflammatory cell infiltration and various degrees of focal myocardial hypertrophy and degeneration (Figures 2A–2C). Transmission electron microscopy (TEM) demonstrated damage to myofibrils and mitochondria, with a blurring of mitochondrial cristae (Figures 2D–2F). The patient recovered sufficiently post-operation. Repeat TTE showed a slightly enlarged RA, intact atrial septum, and normal LVEF (Figure 1D). Chest x-ray revealed a decreased CTR as 0.61 (Figure 1E). At 1-year follow-up, there was a progressive enlargement of the RA (Figures 3A,B), and the patient remained asymptomatic except for growth retardation. Unexpectedly, TTE 2 years after epilepsy surgery demonstrated an enlarged left ventricle (LV) with a left ventricular end-diastolic diameter (LVEDD) of 40 mm, a giant RA (52 mm × 39 mm), a normal PASP of 20 mmHg, and reduced LVEF of 55% (Figures 3C,D). Anti-heart failure therapy was administered, including captopril, diuretics, metoprolol, and acetylsalicylic acid. The patient had a good condition at 5 years post-operation. The size of the RA and LV did not change significantly, and the LVEF was about 50% (Figures 3E,F). Cardiac magnetic resonance (CMR) showed cardiomegaly at the expense of the right atrium with a decreased LVEF of 45% and no typical manifestations of ventricular myocardial fibrosis (Figure 4).

A genetic study showed a heterozygous missense *Pkp2* variant (c.2380T > G; OMIM: 609040; SCV 002576312) related to arrhythmogenic right ventricular cardiomyopathy (ARVC) (Supplementary material S1). Further Sanger sequencing indicated that his mother presented the same variant in *Pkp2*; the variant was classified as a variant of uncertain significance according to the American College of Medical Genetics and Genomics (ACMG) guidelines. Although his mother was asymptomatic, her echocardiography showed an enlarged LA and LV (LVEDD 55 mm), mild to moderate mitral regurgitation, and reduced LVEF (48%) without other cardiac structural defects. Then, the mother was diagnosed with DCM, and the diagnosis of familial DCM was established based on the patient's and his mother's features.

## Discussion

Giant RA is considered to be of congenital origin in the absence of conditions such as congenital heart disease, pulmonary arterial hypertension, or tricuspid valve disease. IDRA is the most common disease mentioned in recent years due to a marked enlarged right atrium, a congenital anomaly with unknown etiology reported in fetuses, infants, children, and adults (2–4). Most patients are asymptomatic, and the most common symptoms are palpitations, dyspnea, and syncope caused by atrial tachyarrhythmias (2). The diagnosis of IDRA is usually established with fetal echocardiography and transthoracic echocardiography, and CT or CMR may be beneficial for a definitive diagnosis (2–4). According to prenatal and postnatal imaging features, IDRA should be considered as the primary disorder in our case. It is important to note that IDRA may be accompanied by congenital heart defects consisting of ASD (5). Our case also has a large secundum ASD, which could have contributed to the enlargement of RA. However, the recurrence of atrial enlargement after surgical repair of ASD suggests that the effect of ASD on giant RA is limited. The postnatal management of asymptomatic patients with IDRA remains controversial. Indications for surgical reduction of RA in children with IDRA have included right atrial thrombus and atrial arrhythmias, as well as others, such as progressive dilatation of the RA, concern about airway compression, and severe tricuspid insufficiency suspected as Ebstein's anomaly (6). So far, many studies suggest that the long-term outcomes of patients post-surgery are also contradictory. Although many cases demonstrate that surgery may indeed relieve symptoms associated with IDRA, some children still suffer the recurrent attack of giant RA (4, 6). Unfortunately, the situation of our case also suggests that the surgical indications for children with IDRA should be more strictly controlled.

In patients with cardiomyopathy, huge atria are commonly seen in restrictive cardiomyopathy (RCM), leading to a significant atrial pressure increase due to limited ventricular diastolic function and progressive atrial enlargement (7). The morphological definition of RCM is based on imaging features demonstrating non-hypertrophied, non-dilated ventricles, with marked bi-atrial enlargement (7). Nevertheless, the findings are not consistent with the characteristics of RCM in this case but with the features of DCM, such as LV dilation and poor cardiac systolic function.

Endomyocardial biopsy (EMB) is the gold standard method for diagnosing acute or chronic inflammatory heart diseases and enables the identification of the underlying etiology of cardiac inflammation (8, 9). Although there are no structural or functional changes in the heart chambers except RA in the early stage of the disease in our case, it is worth noting that the histological findings of damage to LA and RA were present. On the other hand, early involvement of the LV cannot be ruled out despite the absence of an LV biopsy. With the evidence of inflammatory cell infiltration, the probability of inflammatory disorders in the myocardium should still be considered (10).

The diagnosis of familial DCM is confirmed when two or more first-degree relatives have “idiopathic” DCM and/or unexplained death at a young age, and there could be an underlying genetic etiology (11). The pathogenesis of genetically mediated DCM has been associated with genes that encode components of the cytoskeleton, sarcomeric proteins, and nuclear envelope proteins (12). Recently, *Pkp2* gene, encoding components of desmosomes, has also been associated with different inherited cardiac conditions, including ARVC and Brugada syndrome (13). Few studies have suggested that *Pkp2* can also lead to DCM and left ventricular non-compaction (LVNC) (14). However, these associations do not prove causality between *Pkp2* and DCM in our case, and further research should be conducted on the variant we detected.

In summary, DCM with a giant atrium as the first manifestation is an uncommon complex condition. Although the etiology remains unclear, a key factor should contribute to this unique phenotype. This case also reminds us of the importance of good follow-up of children with IDRA, including those after surgery. Further follow-up is needed for this case and his mother to evaluate the prognosis of this phenotype in DCM.

## Data Availability

The original contributions presented in the study are included in the article/Supplementary Material, further inquiries can be directed to the corresponding author.
