# Supplementary material for: Giant right atrium in a child with dilated cardiomyopathy: A case report
Source: Front Cardiovasc Med. 2023 Mar 15;10:1083188. doi: 10.3389/fcvm.2023.1083188 (PMC10050595; doi:10.3389/fcvm.2023.1083188)
Supplement: Supplementary file 1 [file Datasheet1.docx]

Supplementary Material

Giant Right Atrium in a Child With Dilated Cardiomyopathy: A Case Report

Benzhen Wang, Guangsong Shan, Zhen Bing, Qi Zhang, Quansheng Xing, Zipu Li^1*^

*** Correspondence: Zipu Li, Email: apuqd@sina.com**

# Supplementary Data

Supplementary Material should be uploaded separately on submission. Please include any supplementary data, figures and/or tables.

Supplementary material is not typeset so please ensure that all information is clearly presented, the appropriate caption is included in the file and not in the manuscript, and that the style conforms to the rest of the article.

# Supplementary Figures and Tables

For more information on Supplementary Material and for details on the different file types accepted, please see [here](https://www.frontiersin.org/guidelines/author-guidelines#supplementary-material).

## Supplementary Figures


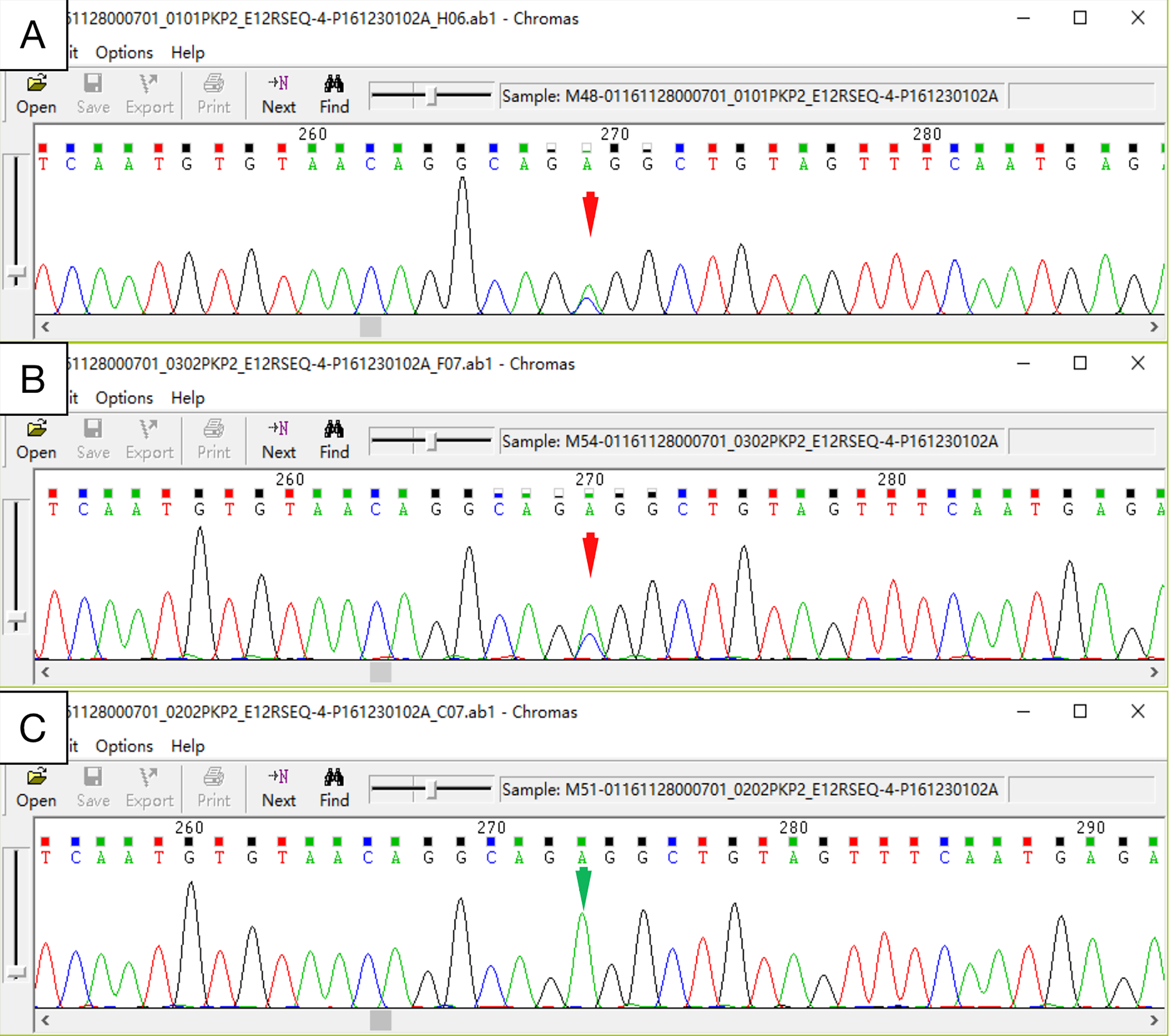


**Supplementary Figure 1.** Sanger DNA sequencing chromatogram detected a heterozygous missense variant (c.2380T>G) of Pkp2 gene in the proband of the patient (A, red arrow) and his mother (B, red arrow) but not his father (C, green arrow).


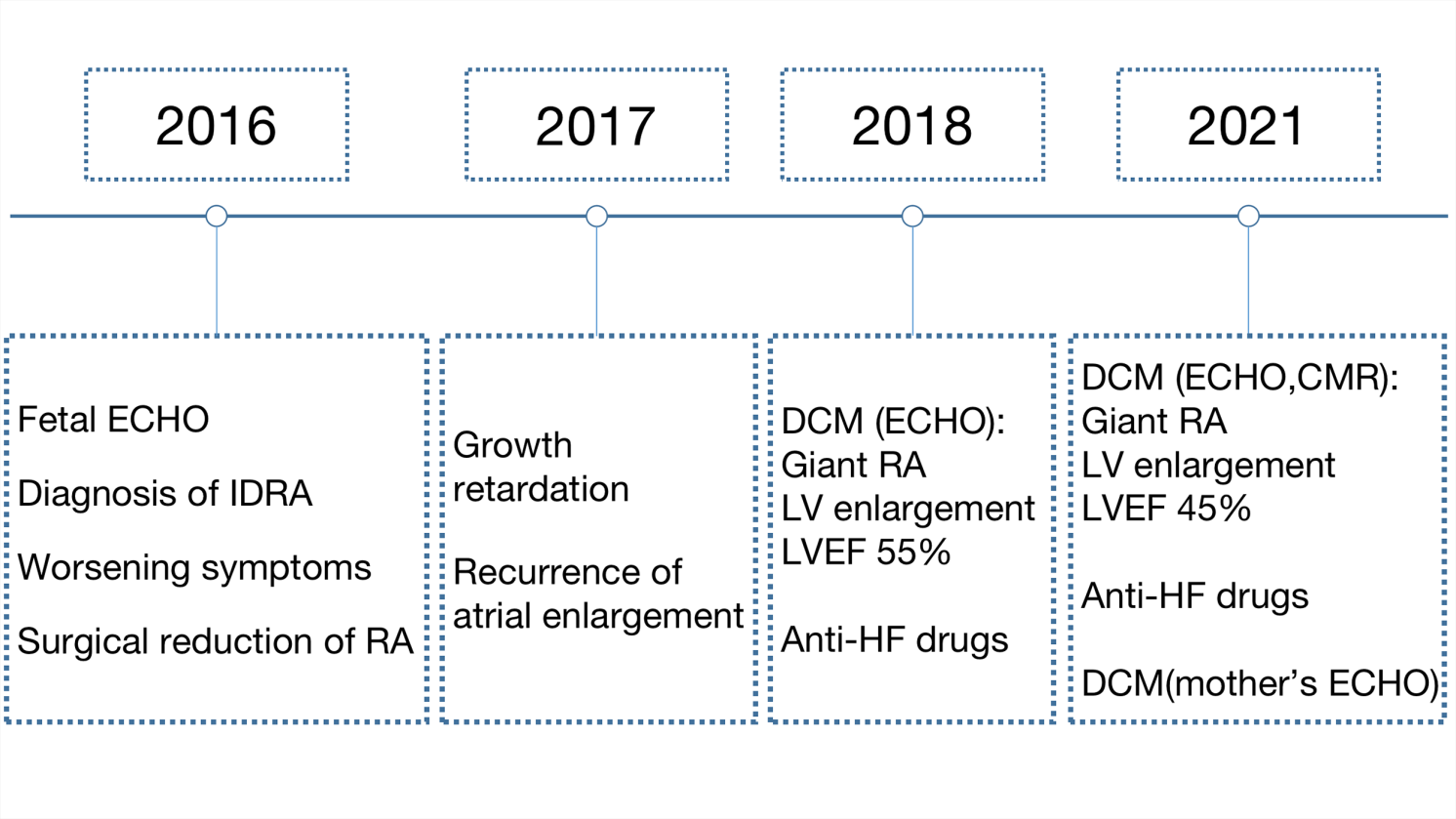


**Supplementary Figure 2.** Timeline. The relevant data of the patient is displayed in the timeline. ECHO echocardiography; IDRA idiopathic dilatation of the right atrium; RA right atrium; LV left ventricle; LVEF left ventricular ejection fraction; HF heart failure.
