# Supplementary figures and images for: Giant right atrium in a child with dilated cardiomyopathy: A case report
Source: Front Cardiovasc Med. 2023 Mar 15;10:1083188. doi: 10.3389/fcvm.2023.1083188 (PMC10050595; doi:10.3389/fcvm.2023.1083188)

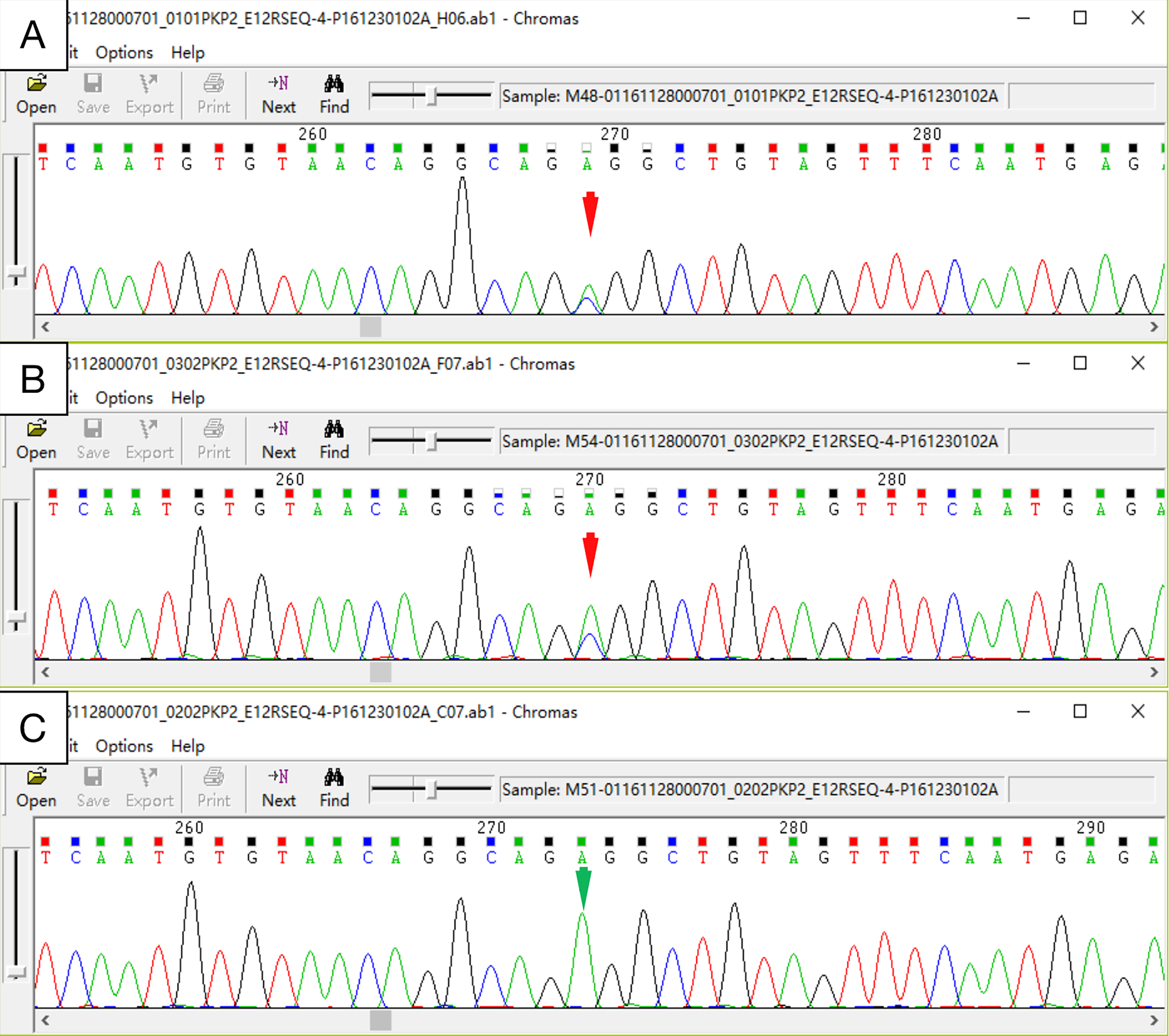

Supplement: Supplementary file 2 [file Image1.tif]

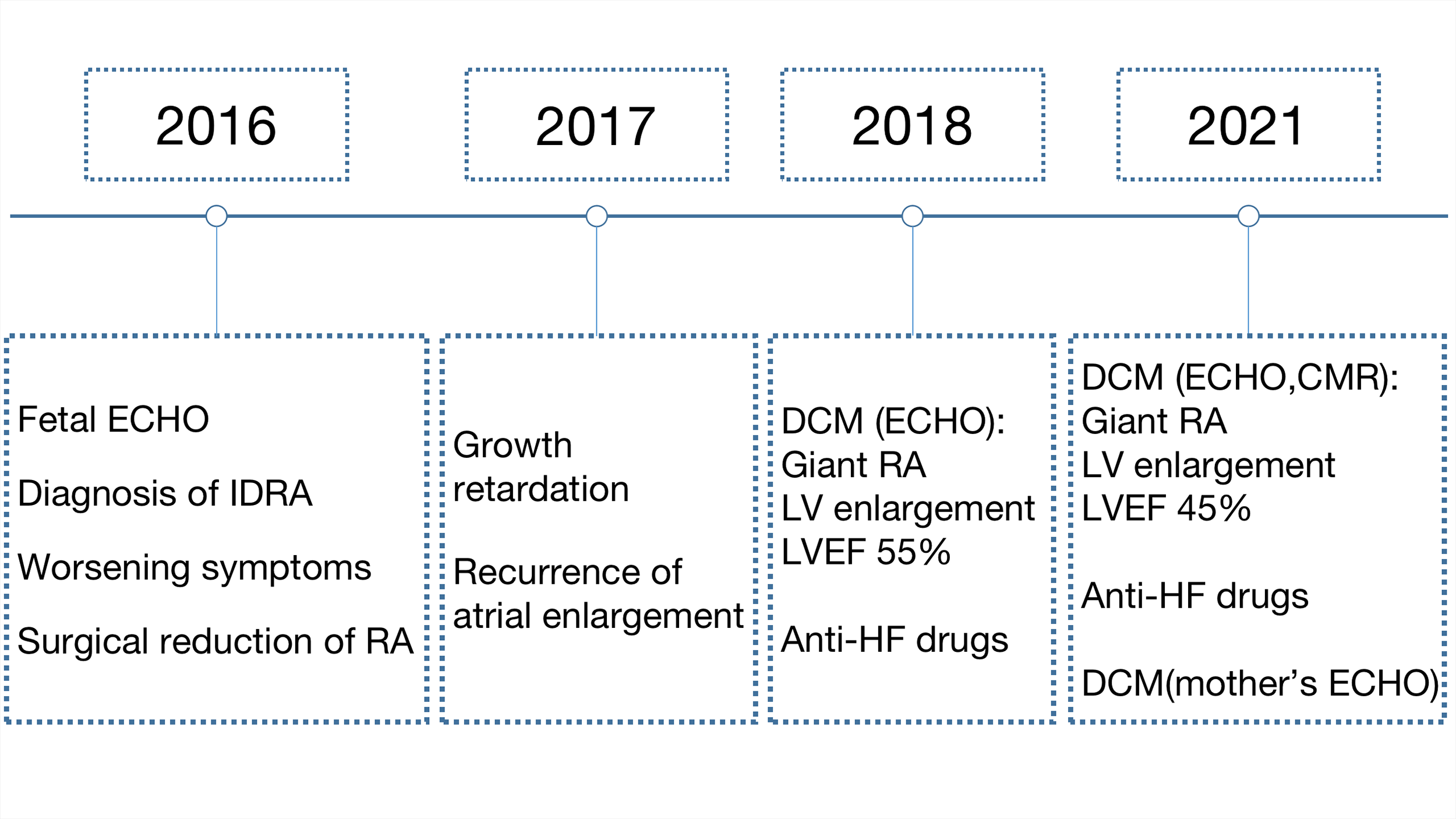

Supplement: Supplementary file 3 [file Image2.tif]
